# Determinants of MRSA carriage/infection in health care workers in the Netherlands

**DOI:** 10.1186/1753-6561-5-S6-P12

**Published:** 2011-06-29

**Authors:** M van Rijen, J Kluytmans, CAM-study Group

**Affiliations:** 1 Laboratory for Microbiology and Infection Control, Amphia Hospital, Breda, Netherlands

## Introduction / objectives

Nosocomial transmission is considered to be the most important source for MRSA colonisation of health care workers (HCW). However, the epidemiology is changing and community-acquired MRSA is increasing. The objective of this study was to investigate the determinants of MRSA carriage/infections in HCW in The Netherlands.

## Methods

All newly identified HCW with MRSA in seventeen hospitals in the Netherlands were included from January 2009 until December 2010. MRSA determinant analysis was done based on the HCW’s history combined with molecular typing results and then classified in risk groups described in the national infection prevention guidelines.

## Results

In two years 68 HCW were found to be MRSA positive for the first time. Analysis of risk factors revealed that 27.9% of the HCW (n=19) were considered to be caused by nosocomial transmission, 19.1% (n=13) had been exposed to pigs/veal calves, 10.3% (n=7) had worked in a foreign hospital, 1.5% (n=1) were colonised due to transmission in a psychiatric home and 41.2% (n=28) could not be classified in a known risk group. Based on spa-typing, Livestock Associated MRSA (ST-398) was found in 100% of the HCW who had been exposed to pigs/veal calves (n=13), in16.7% of the HCW who had worked in a foreign hospital (1 positive, 5 negative, 1 unknown) and in 15.8% of the HCW who were colonised due to nosocomial transmission (3 positive, 16 negative) . Remarkably, 22.2% of the strains in the group with unknown determinants belonged also to this clonal complex (6 positive, 21 negative, 1 unknown).

## Conclusion

The majority (n=28) of newly identified MRSA positive HCW reported no known source. Strains with spa-types indicative for Livestock Associated MRSA were found in 18.9% of individuals who did not report contact to livestock. This indicates that LA-MRSA is spreading, in the community and/or in the hospital.

## Disclosure of interest

None declared.

